# Perceptions of Maternal Discrimination and Pregnancy/Postpartum Experiences Among Veterinary Mothers

**DOI:** 10.3389/fvets.2020.00091

**Published:** 2020-03-06

**Authors:** Annie S. Wayne, Megan K. Mueller, Marieke Rosenbaum

**Affiliations:** ^1^Department of Clinical Sciences, Cummings School of Veterinary Medicine at Tufts University, North Grafton, MA, United States; ^2^Jonathan M. Tisch College of Civic Life, Tufts University, Medford, MA, United States; ^3^Department of Infectious Disease and Global Health, Cummings School of Veterinary Medicine, Tufts University, North Grafton, MA, United States

**Keywords:** maternal, discrimination, gender, fertility, postpartum depression, inequity

## Abstract

**Objective:** To describe perceptions of maternal discrimination and to begin to understand patterns around timing of starting families, infertility, and post-partum depression among veterinary mothers.

**Design:** Cross-sectional questionnaire with closed and open-ended questions posted to a social media platform “Moms with a DVM.”

**Sample:** 1,082 veterinary mothers in the United States.

**Procedures:** An online questionnaire was administered regarding perceived discrimination, inequities in the work-place due to pregnant or maternal status, desired accommodations, timing of pregnancy(ies), fertility issues, and postpartum experiences.

**Results:** At least one form of perceived discrimination was reported by 819 (75.7%) respondents (*M* = 2.6, *SD* = 2.1, range 0–10). Specifically, 789 (72.9%) reported maternal discrimination. Over half of the sample (*n* = 632, 58.4%) reported at least one instance of perceived inequity in the workplace due to status as a mother (*M* = 1.23, *SD* = 1.4, range 0–5). A majority (906, 83.7%) reported that their career had “definitely” or “maybe” affected the timing of their children. One hundred eighty-nine respondents (17.5%) experienced at least one miscarriage, and 192 (17.6%) used fertility treatment due to difficulty conceiving. Postpartum depression was diagnosed in 181 respondents (16.7%), and 353 (32.6%) reported symptoms consistent with postpartum depression but did not seek medical care. Of 953 participants who needed accommodations for breastfeeding and/or pumping while at work, 130 (13.6%) reported excellent accommodations, 454 (47.6%) adequate, 258 (27.1%) inadequate, and 111 (11.6%) had no accommodations provided.

**Conclusions and Clinical Relevance:** Participants reported experiences of perceived maternal discrimination, as well as inequities and lack of support services due to status as a mother. These results highlight the need for attention and changes to ensure veterinarians have supportive and sustainable career options.

## Introduction

In the last 60 years, veterinary medicine has shifted from a male-dominated (nearly 90%) to a mostly female-dominated (about 80%) profession (1). Despite these demographic changes, female veterinarians are still paid less than their male counterparts, have a higher debt to income ratio (2) and experience gender discrimination (3). The number of women in the United States becoming mothers has increased over the last 20 years and in 2016, 86% of women were also mothers by the end of their childbearing age (4). A majority (51%) of working women in the United States say that having children has “made it harder for them to advance” in their career compared to 16% of men (4). In particular, women in science, technology, engineering, and math (STEM) careers specifically experience discrimination and face challenges navigating parenting and demanding careers. One recent study found that young parents were more likely to leave full time employment in STEM careers compared to their non-parent counterparts and that mothers left at twice the rate of fathers (5). A recent survey of physician mothers found that 66.3% perceive gender discrimination and 35.8% perceive discrimination based on their pregnant or maternal status at work (6).

Navigating the challenges of parenting and pursuing a veterinary career contributes to overall wellness among veterinary professionals. Previous research by the authors shows that parental support by veterinary schools and training programs is lacking and that many trainees perceive that having children during their training years (veterinary school, internships and residency training programs) is not feasible (7, 8). There is currently no data regarding maternal discrimination and the effects it may have on veterinarian mothers. The goal of this research was to explore perceived discrimination among veterinary mothers in the United States and was modeled after a study of physician mothers (6) to compare experiences with a similar population. An additional goal was to look at baseline data on decisions to start a family, infertility and post-partum depression in veterinary mothers in order to inform and direct future research in this area.

## Materials and Methods

### Study Design and Overview

This study was cross-sectional in design and used an online anonymous questionnaire, composed of both closed and open-ended questions, that was posted to a social media platform closed group “Moms with a DVM.” Questions were designed to mirror data presented in the Journal of the American Medical Association (JAMA) (6) that investigated perceived rates of discrimination among physician mothers so we could compare their results to the experiences of veterinary mothers. Additional questions about infertility, workplace accommodations for parenting, and postpartum depression were added. Participants were eligible if they were over the age of 18 years, identified as a mother or pregnant, and had received a DVM or equivalent degree and lived in the United States. The research was reviewed and granted exempt status from the Tufts University Social, Behavioral, and Educational Research Institutional Review Board. The survey was administered by Qualtrics and was posted to the group three times between Nov 28 and Dec 10, 2018, with additional posts to sub-groups in the same time-frame. “Moms with a DVM” had over 10 thousand members at that time with approximately 200 new posts per day.

### Survey

Inclusion criteria selected participants who were members of the group “Moms with a DVM” who were over 18 and who self-identified as pregnant or a mother. The questionnaire was composed of closed-ended questions to obtain the following data: demographic information, number and age of children, level of post-veterinary training obtained, type of current employment, whether participants had “ever felt discriminated against” based on 11 factors: their gender, maternal status, being pregnant or breast-feeding, taking maternity leave, race, ethnicity, age, sexual orientation, mental health status, or physical disability [derived from Adesoye et al. (6) study assessing maternal workplace discrimination in physicians]. Additionally, participants were asked about inequities in the workplace due to their maternal status: pay or benefits not equal to peers, not fairly considered for promotion or senior management, treated with disrespect by support staff, held to a higher performance standard than peers, and not included in administrative decision making (6). Participants were asked to select the top three workplace changes that would be most important “to you as a mother” from a set list. Options included: more flexible weekday schedule, higher pay, longer paid maternity leave, option to work part-time, support with home services, childcare onsite, backup childcare, option to not work on weekends, more vacation days, option to not take on-call, flexibility to work from home, additional support for breastmilk pumping, more sick days, and other (6). In addition, participants were asked about support and accommodations for breast-feeding or pumping, a question about how career choices influenced timing of pregnancy(ies), and if mothers experienced any infertility issues or post-partum depression. Finally, there was a space for open comments on any aspect of maternal discrimination.

### Data Analysis

Descriptive statistics and frequencies were calculated using statistical software^1^. To evaluate associations between demographic variables and material discrimination, adjusted logistic region models were used to estimate odds ratios and 95% confidence intervals adjusting for age and race/ethnicity (6). For sexual orientation and race/ethnicity, descriptive categories were collapsed into binary variables for the regression analysis since sample sizes in the non-majority individual categories were low (see Table 1).

**Table 1:** Demographic characteristics of the survey respondents and maternal discrimination.

	**Respondents, n (%)**			
**Characteristic**	**Total *n* = 1,082**	**Experienced maternal discrimination *n* = 789**	**OR (CI) for experiencing maternal discrimination**	***P* value**
**AGE IN YEARS**				
≤30	123 (11.4)	85 (69.1)	Reference	
31–35	388 (35.7)	278 (71.6)	1.1 (0.7–1.7)	0.596
36–40	384 (35.5)	289 (75.3)	1.3 (0.9–2.1)	0.190
41–45	111 (10.3)	84 (75.7)	1.4 (0.8–2.5)	0.282
≥46	76 (7.0)	53 (69.7)	1.0 (0.5–1.9)	0.924
**RACE/ETHNICITY**				
Non-Hispanic White	1020 (94.3)	740 (72.6)	Reference	
Non-Hispanic Black	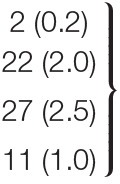	49 (79.0)	1.4 (0.7–2.6)	0.306
Asian				
Hispanic				
Other or prefer not to say?				
**SEXUAL ORIENTATION**				
Straight/heterosexual	1069 (98.8)	780 (73.0)	Reference	
Gay/lesbian/homosexual	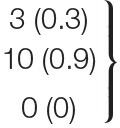	9 (69.2)	0.9 (0.3–3.0)	0.308
Bisexual				
Transsexual				
**MARITAL STATUS**				
Not currently married, never	28 (2.6)	22 (78.6)	Reference	
married, divorced, widowed				
Married	1021 (94.3)	742 (72.7)	0.7 (0.3–1.8)	0.478
Partnered but not currently married	33 (3.0)	25 (75.8)	0.8 (0.2–2.8)	0.743
**NUMBER OF CHILDREN**				
0	14 (1.3)	11 (78.6)	Reference	
1	411 (37.6)	285 (69.3)	0.6 (0.1–2.1)	0.389
2	483 (44.1)	364 (75.4)	0.7 (0.2–2.8)	0.662
3	154 (14.1)	113 (73.4)	0.7 (0.2–2.6)	0.572
>3	32 (2.9)	26 (81.2)	1.1 (0.2–5.3)	0.9
**AT LEAST 1 CHILD** **<6 YEARS**				
No	161 (14.9)	115 (71.4)	Reference	
Yes	921 (85.1)	674 (73.2)	1.1 (0.7–1.8)	0.551
**CURRENTLY PREGNANT OR GAVE BIRTH IN THE LAST YEAR**				
No	753 (69.6)	560 (74.4)	Reference	
Yes	329 (30.4)	229 (69.6)	0.8 (0.6–1.1)	0.232
**PRACTICE TYPE**				
Small animal clinical	775 (71.6)	549 (70.8)	Reference	
Large animal clinical	63 (5.8)	52 (82.5)	2.0 (1.0–3.9)	0.043^*^
Mixed animal clinical	86 (7.9)	68 (79.1)	1.6 (1.0–2.9)	0.072
Laboratory animal/public	158 (14.6)	120 (75.9)	1.3 (0.8–1.9)	0.25
sector/other				
**EMPLOYER**				
Private company/small business	909 (84.1)	664 (73.1)	Reference	
Academic	173 (16.0)	125 (72.3)	0.9 (0.7–2.6)	0.7
institution/government/other				
**POST–DVM TRAINING**				
Current student/intern/resident	17 (1.6)	14 (82.3)	Reference	
Internship trained	177 (16.4)	132 (74.6)	0.6 (0.2–2.3)	0.494
Residency trained	100 (9.2)	72 (72.0)	0.5 (0.1–2.0)	0.367
No internship or residency	788 (72.8)	571 (72.5)	0.6 (0.2–2.1)	0.413
completed				

* *Denotes statistical significance of P < 0.05*.

Qualitative data collected in the open comments section were managed using a qualitative data analysis software tool^2^. Responses were sorted into themes where each response could be tagged in as many thematic categories as appropriate.

## Results

A total of 1,160 respondents participated in the survey. There was a response rate of approximately 10% based on total number of members in the group. Four surveys were removed for not meeting inclusion criteria, and 74 were removed for incomplete quantitative data (only participants with complete questionnaires were retained), leaving an analytic sample of 1,082 participants. Age of the participants ranged from 24 to 71 years old, *M* = 36.3, *SD* = 5.1; demographic characteristics are listed in Table 1.

**Figure 1:**
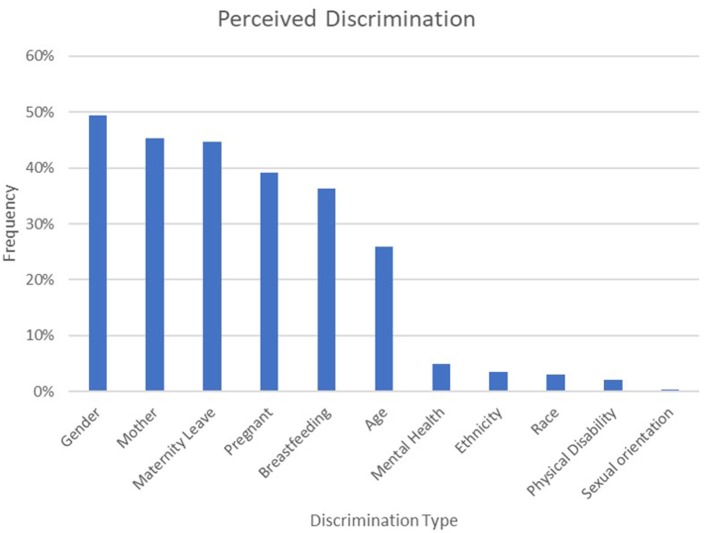
Perceived discrimination among 1,082 survey respondents. Respondents could select multiple types of perceived discrimination.

Of the 1,082 respondents, 819 (75.7%) reported experiencing at least one form of perceived discrimination (*M* = 2.6, *SD* = 2.1, range 0–10), see Figure 1. There was overlap between maternal and non-maternal discrimination with 317 (29.3%) participants reporting both types. Likelihood of experiencing maternal discrimination did not vary significantly by the demographic variables, although veterinarians who worked in large animal practice were more likely to have experienced discrimination (Table 1).

**Figure 2:**
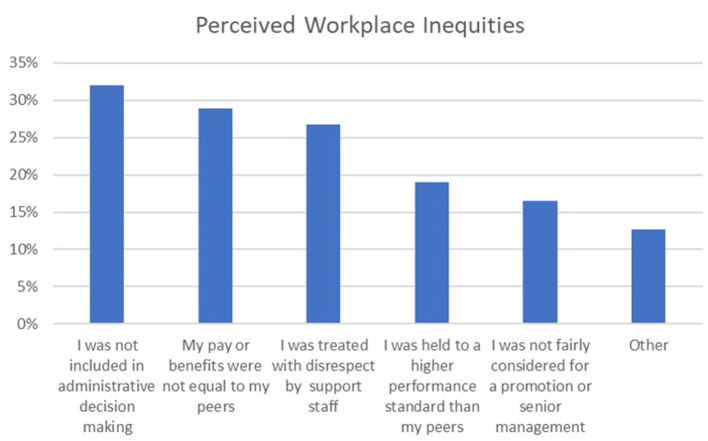
Perceived inequity by 1,082 survey respondents. Respondents could select multiple types of perceived discrimination.

Over half of the sample (*n* = 632, 58.4%) reported experiencing at least one instance of perceived inequity in the workplace due to status as a mother (*M* = 1.23, *SD* = 1.4, range 0–5) (Figure 2). Specifically, 346 (32%) reported not being included in administrative decision making, 312 (28.8%) reported having pay or benefits not equal to peers, 289 (26.7%) were treated with disrespect by support staff, 206 (19.0%) felt they were held to a higher performance standard than peers, and 179 (16.5%) felt they were not fairly considered for a promotion or senior management position due to their status as a mother.

Participants were asked to report the top three workplace changes that would make a difference to them as a mother: 602 (55.6%) selected a more flexible weekday schedule, 544 (50.3%) longer paid maternity leave, 324 (29.9%) childcare availability onsite, 298 (27.5%) having the option to not work on weekends, 288 (26.6%) having the option to work part-time, 248 (22.9%) higher pay, 224 (20.7%) having the option to not take on-call, 209 (19.3%) backup childcare, 159 (14.7%) additional support for breastmilk pumping, 119 (11.0%) more vacation days, 97 (9.0%) flexibility to work from home, 107 (9.9%) more sick days, 15 (1.4%) support with home services, and 9 (0.8%) would like other changes.

A majority of the sample (906, 83.7%) reported that their career had “definitely” or “maybe” affected the timing of their children. Maternal age at the time of first child ranged from 18 to 44 years (*M* = 31.2; *SD* = 3.7). With regard to fertility, 189 (17.5%) of the sample experienced at least one miscarriage, and 192 (17.6%) used fertility treatment due to difficulty conceiving. During the postpartum period, 181 (16.7%) experienced diagnosed postpartum depression, and 353 (32.6%) reported symptoms but no diagnosis, yielding a total of nearly 50% of the study population who experienced symptoms of postpartum depression. See Table 2 for full descriptive results regarding fertility, pregnancy, and postpartum experiences. Of the 953 participants who needed accommodations for breastfeeding and/or pumping while at work, 130/953 (13.6%) reported their accommodations as excellent, 454/953 (47.6%) as adequate, 258/953 (27.1%) as inadequate, and 111/953 (11.6%) had no accommodations provided by their workplace. Of the 521 individuals who needed breastfeeding and/or pumping accommodations at continuing education or a conference, 24/521 (4.6%) reported available accommodations as excellent, 152/521 (29.2%) reported them as adequate, 189/521 (36.3%) as inadequate, and 156/521 (29.9%) reported experiencing no availability of accommodations.

**Table 2:** Frequency of experiences with premature birth, miscarriage, fertility treatment, and postpartum depression among 1,082 veterinary mothers administered a questionnaire through the closed, online group “Moms with a DVM.”

**Respondent experience**	***n*(%)**
Experienced a premature birth	167 (15.4%)
**EXPERIENCED MISCARRIAGE**	
No	726 (67.1%)
One	241 (22.3%)
Two	74 (6.8%)
More than two	35 (3.2%)
I prefer not to say	6 (0.6%)
Fertility treatment due to difficulty conceiving	192 (17.6%)
**POSTPARTUM DEPRESSION (PPD)**	
Yes, diagnosed and treated by a medical professional	181 (16.7%)
Symptoms but not diagnosed by a medical professional	353 (32.6%)
No	531 (49.1%)
Not applicable (currently pregnant)	13 (1.2%)
I prefer not to say	4 (0.4%)
**CAREER CHOICES AFFECTED TIMING OF PREGNANCY(IES)**	
Definitely yes	731 (67.6%)
Maybe yes	175 (16.2%)
Not sure	22 (2.0%)
Probably not	92 (8.5%)
Definitely not	62 (5.7%)

### Open-Ended Responses

There were a total 269 meaningful responses to the question open to any comments on maternal discrimination or challenges in the workplace due to status as a parent. Comments that included “none,” “N/A” or an incomplete thought were excluded. Comments that illustrate the range of responses for each category are provided in Table 3. Sixty-three responses (23.4%) were coded as “sexist, discriminatory or disrespectful comments made by staff due to maternal or pregnant status.” There were 54 responses (20.1%) regarding pay or promotion status. Of these responses, 20/54 (37.0%) describing losing a job due to maternal or pregnancy status, 14/54 (25.9%) described pay or status (full-time vs. part-time) was negatively impacted by maternal or pregnancy status, 10/54 (18.5%) said their promotion status was negatively impacted based on pregnancy or maternal status, 10/54 (18.5%) described being discriminated against during an interview process due to future or current maternal or pregnancy status, and 10/54 (18.5%) said they were not hired for a job due to pregnant or maternal status. There were 53 comments (19.7%) on issues of time pressure related to childcare and working status; 22/53 (41.5%) described difficulties around lack of a flexible schedule related to securing childcare, 18/53 (33.9%) described lack of ability to take time off to care for sick children, and 11/53 (20.8%) described other types of challenges around childcare and working. Forty-six respondents (17.1%) commented on lack of adequate leave time and/or pay. Thirty-six respondents commented on lack of appropriate time (20/36; 55.6%) or lack of appropriate space (14/36; 38.9%) for pumping. Sixteen respondents (5.9%) commented on safety issues during pregnancy; 12/16 (75%) said they had inadequate accommodations and 4/16 (25%) said they felt unsafe during their pregnancies. Five respondents (1.9%) said they regretted their choice to be veterinarians and/or were actively looking to leave the profession. Eighteen (6.7%) had positive comments and 38 (14.1%) were categorized as “other.”

**Table 3:** Representative comments from 269 meaningful responses to the open-ended question regarding maternal discrimination or challenges in the workplace due to status as a parent.

**Comment type**	**Representative comment(s)**	**Number of comments**
Sexist, discriminatory or disrespectful comments due to maternal or pregnant status	Office manager commented that we should only hire male vets in the future so they don't leave to start a family. I have had clints choose other Drs since I am not as a available after office hours. I devote that time to family. A client actually told me she was appalled I chose to be a mom and a vet. She felt I couldn't do that as a vet since my primary duty should be to my patients as a vet and not my kids.	63
Pay or promotion negatively impacted or loss of job or not hired due to maternal or pregnancy status	I was fired from my last job 2 days before returning from maternity leave. I was replaced by the doctor I recommended to cover my maternity leave. I had been the only associate at practice for 7 years and no problems or anything other than praise until I announced my pregnancy. I watched 4 support staff get fired while pregnant or on maternity leave prior to me being fired. I was not considered for partner even though I was a high producer and had a large client base. When I asked my boss for consideration he flat out to my face said no because I chose the family track. At an interview, a male owner told me that I could never be a good vet and a good mom.	54
Difficulties around lack of a flexible schedule related to securing childcare	My Chiefs of Staff were fine with schedule modifications for employees to care for their own pets, yet considered it unfair if I needed to leave at a certain time to meet the school bus or worked fewer nights than the other associates (even though I had reduced pay due to these scheduling necessities to provide care for my child).	22
Lack of ability to take time off to care for sick children	The few times my child has been sick, I have been unable to care for her adequately due to lack of support from my job to help find coverage.	18
Other types of challenges around childcare	I requested to move my lunch break to the afternoon to pick up my kids from school, am so was told that I was “stealing company time” when I was simply moving the hour provided to me for lunch.	11
Lack of adequate leave time and/or pay	The biggest struggle as a mother was the length of maternity leave: only 6 weeks and unpaid. I work at a small practice, so being short a vet is tough for my coworkers, but 6 weeks was not enough time home with my baby!	46
Lack of appropriate time or lack of appropriate space for pumping	I'm having problems finding the time to pump as I'm not allowed to block out time, and when we get busy, that ends up dropping to the way side. I am also expected to answer phones and write charts while I pump, and therefore can never get a good letdown like I get at home, so I end up engorged and sore at the end of every day. My staff sees me pumping as an inconvenience and gets huffy when I ask them to finish things up while I go pump. I was shamed for pumping at work. I was told it was disgusting and reprimanded for washing my pumping equipment at work after pumping. I pumped in a supply closet with chemotherapeutic waste!	36
Inadequate safety accommodations	Unsafe radiation practices continued although I requested they end (rads taken without warning while unshielded people were in the way).	16
Regretted their choice to be veterinarians and/or were actively looking to leave the profession	I am actively seeking to leave the profession. The stress, lack of adequate pay, and time I am required to spend away from my children is not worth it. By the time I can get home from my job, there is minimal to no time to interact with my children. I am seeking to completely leave veterinary medicine. It has been detrimental to my mental, financial, and physical health.	5
Positive	I was working in a corporate hospital while pregnant and pumping, and I was treated with respect and given the time I needed. My short-term disability and generous PTO helped pay for most of my 12 weeks maternity leave. I have been very lucky to have a supportive male boss who allowed me with no complaints 3 months of unpaid maternity leave, pumping accommodations, and the freedom to pick the days and hours I wanted to work part time. It has made returning to work very manageable and he has beyond earned my loyalty as an associate to stay indefinitely with the practice.	18
Other	My work was supportive - more invasive comments from clients. Previous employer (equine private practice) asked that I give a three-year verbal commitment to not having a baby when I joined the practice.	38

## Discussion

In this anonymous survey of veterinarians who are also mothers, the vast majority (about 75%) reported experiencing at least one type of perceived discrimination, with nearly 73% of respondents reporting discrimination based on their maternal status. In addition, more than half of respondents reported perceived inequity based on their maternal status. Although these responses targeted a specific social media group, a subjective description of the group is an inclusive, supportive and diverse group of women that offer support and advice on a wide range of topics both professional and personal. These data were from a small group of women who likely have an interest in this topic, however, the responses indicate that maternal discrimination and other issues for veterinary mothers are problematic, deserve additional research with more robust methodology and should prompt discussion of systemic institutional changes in the profession. Given that the veterinary profession is now largely made up of women (1), the widespread perceived discrimination likely has far reaching and long-lasting impacts for the profession. As has been demonstrated in the human medicine literature (6), perceived discrimination may impact rates of burnout, retention and career satisfaction in addition to impacting earning power.

Overall frequency of perceived discrimination among veterinarian mothers as compared to a similar survey of physician mothers were similar: 75.7% of veterinarians and 77.9% of physicians experienced discrimination of any type (6). However, in our study, 72.9% of veterinarians reported perceived maternal discrimination as compared to 35.8% of physician mothers responding to a similar survey (6). Discrimination based on gender demonstrated a reverse pattern, with 39.1% of veterinarians reporting perceived discrimination and 66.3% of physician mothers (6). One possible explanation is the higher percentage of women in veterinary medicine as compared to human medicine (in 2017, 80.5% of matriculating veterinary school were women, compared to 50.7% of medical school students) (1, 9) influences the prevalence of gender discrimination. Compared to veterinary medicine, in which the first published papers exploring the social and cultural implications of the increasingly female workforce began to emerge in the late 90s (10) and the first paper focusing on parenting was published in 2018 (7), attention to the struggle of female physicians dates back to the late 70s (11) and attention to the struggle physician mothers face as they balance dual roles (parenting and being a physician) dates back to the late 90s (12). It is possible that the human medical profession has dedicated more attention to this issue dating further back, which has resulted in increased awareness and in lower rates of perceived maternal discrimination in physicians as compared to veterinarians. Regardless of the differences between perceived maternal discrimination among veterinary and physician mothers, the high prevalence of perceived discrimination in the workplace in both populations is significant and warrants attention as the professions work to improve wellness.

The top three ranked accommodations desired by veterinary mothers were flexibility in the workday schedule, longer paid maternity leave, and childcare onsite. According to a recent survey of veterinarians by DVM 360, 64% of women and 42% of men would take less pay for more flexibility in working hours, highlighting the importance of flexibility in the workforce (13). Our results suggest that employers could improve job satisfaction by prioritizing flexibility for parents in the workplace. More research into types of flexibility that are desired by parents (i.e., can leave for an extended lunch break to visit child, taking a weekday off as needed, revisiting schedule yearly as parenting roles change with age) and the feasibility and management systems that can be applied to provide flexibility are needed. This may differ by workplace setting and this data is skewed toward small animal veterinarians. Additional research to further describe accommodations desired and possible in different settings would be needed to help guide any future recommendations.

Nearly 84% of respondents reported that timing of children was definitely or maybe influenced by their career choices. Recent literature found similar results among veterinary surgeons and found that women delay childbearing for longer than men (14). Given that the profession is predominantly made of women and childbearing age overlaps with veterinary training and early career building phases for most people, this is unsurprising. In this study, over 30% of respondents said they had experienced at least one miscarriage, which is higher than nationally reported rates of 8–20% (15). Reasons for the higher rates are unknown, but delaying pregnancy due to career choices and/or lack of accommodations and unsafe workplace environments may be contributing factors, as it is widely accepted that veterinarians face numerous hazards to reproductive health in the workplace (16). This study also showed higher rates of fertility treatment (17.6%) as compared with national rates (12%) (15), and higher rates of self-reported post-partum depression (over 30% in this study as compared with about 10% reported by CDC) (17, 18). However, sub-clinical depression is underexplored, and should be an important component of future research in this area. Infertility has previously been shown to evoke distress, anxiety, and feelings of failure, loss and pain (19). This initial survey of veterinary mothers indicate that rates of infertility, and as a result stress associated with infertility, may be higher among the veterinary profession, contributing to recent literature and commentary on mental health in the veterinary profession. Additional data to determine if this is true across more diverse samples of female veterinarians is needed. The higher rate of fertility treatment observed among our sample may be associated with intentional delays in starting a family among the profession due to the perception that it is not feasible to do both at the same time (7), however more research is needed to determine the drivers of fertility treatment among veterinary women, as well as the financial burden of fertility treatment on a profession known to be plagued by high student debt upon graduation.

Veterinarians who worked in large animal practice were more likely to have experienced discrimination than veterinarians in other specialties. A recent study found that among veterinary surgeons, large animal private practitioners worked longer hours and had the most on-call responsibility, and that women earned less than men in this field even after adjusting for all relevant covariates (20). In another study of veterinary surgeons, the same group found that women in large animal practice were less likely to be married, in a domestic partnership, and to have children compared to women in small animal practice (14). Collectively these findings indicated that there are differences in work-culture regarding gender dynamics among subspecialties in veterinary medicine, and that issues surrounding gender equity and maternal discrimination warrant closer attention—and provide an opportunity for meaningful intervention—across the profession.

Women in veterinary medicine (14, 20) and STEM professions in general are adversely affected in terms of their earning power and having children may widen the gap. “Even mothers who remain in the professional workforce full time encounter stereotypes painting them as less competent than equally qualified men and childless women, and face salary penalties and career barriers even while contributing the same dedicated work” (5). Maternal discrimination and lack of perceived support for veterinarians who also are parenting contributes to the mental health load and stress of many.

This survey was a convenience sample administered through a Facebook group and limitations include a lack of diversity among respondents, possible selection bias and small sample size. Additional studies are needed to determine if these data are replicable in a larger population of veterinary mothers in the US. Despite these limitations, the high frequency of perceived discrimination among veterinarian mothers should be considered when thinking about the future of the profession and how to support current veterinarians.

Recently, an article with a description of parental leave policies during medical training was published and included a call to action in the medical profession (21). The results from this study and prior related work (7, 8) support the need for similar recommendations in the veterinary profession and indicate that veterinarians want changes. Qualitative comments from participants in this survey said “I feel like we are still in the dark ages. I faced discrimination when all three [of my] children were born and it has continued. My children were referred to as parasites. My maternity leaves were considered hardships for my co-workers. The other women without children I work with are resentful and have continued to insinuate I don't work as hard [as they do] due to my children.” “During veterinary school one of the doctors in the clinic during fourth year told me that I could choose to be a mother or a doctor, but I couldn't do both effectively. She was a woman. I'll never forget how that statement made me feel as I already had two children. It was terribly deflating.” The real changes needed to accommodate all veterinarians who also wish to be parents and have work-life balance are far reaching and require commitment at all levels of training and employment. In order to continue to attract top level talent and to create successful long-term careers, the professional organizations should consider implementing changes that support veterinary mothers (and fathers).

The findings from this study support the need for future research in this area to further encourage changes to the profession that support veterinarian mothers and fathers as well as to further describe the ways in which maternal and gender discrimination impact the profession and how changes can be incorporated into veterinary medicine in a sustainable way.

## Data Availability Statement

The datasets generated for this study are available on request to the corresponding author, pending IRB approval.

## Ethics Statement

The research was reviewed and granted exempt status from the Tufts University Social, Behavioral and Educational Research Institutional Review Board. The ethics committee waived the requirement of written informed consent for participation.

## Author Contributions

AW, MM, and MR contributed to the design of the survey. AW and MR distributed the survey and reminders to closed groups via social media platform. MM and MR performed quantitative data analysis. AW compiled and sorted qualitative data. AW, MM, and MR contributed to writing of the final manuscript.

### Conflict of Interest

The authors declare that the research was conducted in the absence of any commercial or financial relationships that could be construed as a potential conflict of interest.

## Footnotes

^1^SPSS version 25 and Stata/IC version 14.

^2^NViVo 12.3.0.
